# Heuristic machine learning approaches for identifying phishing threats across web and email platforms

**DOI:** 10.3389/frai.2024.1414122

**Published:** 2024-10-21

**Authors:** Ramprasath Jayaprakash, Krishnaraj Natarajan, J. Alfred Daniel, Chandru Vignesh Chinnappan, Jayant Giri, Hong Qin, Saurav Mallik

**Affiliations:** ^1^Department of Information Technology, Dr. Mahalingam College of Engineering and Technology, Pollachi, India; ^2^School of Computer Science and Engineering, Vellore Institute of Technology, Vellore, India; ^3^Department of Computer Science and Engineering, Karpagam Academy of Higher Education, Coimbatore, India; ^4^Department of Mechanical Engineering, Yeshwantrao Chavan College of Engineering, Nagpur, India; ^5^Department of Computer Science and Engineering, University of Tennessee, Chattanooga, TN, United States; ^6^Department of Computer Science, School of Data Science, Old Dominion University, Norfolk, VA, United States; ^7^Department of Environmental Health, Harvard T H Chan School of Public Health, Boston, MA, United States; ^8^Department of Pharmacology and Toxicology, University of Arizona, Tucson, AZ, United States

**Keywords:** email, URL, website, phishing, social engineering attacks

## Abstract

Life has become more comfortable in the era of advanced technology in this cutthroat competitive world. However, there are also emerging harmful technologies that pose a threat. Without a doubt, phishing is one of the rising concerns that leads to stealing vital information such as passwords, security codes, and personal data from any target node through communication hijacking techniques. In addition, phishing attacks include delivering false messages that originate from a trusted source. Moreover, a phishing attack aims to get the victim to run malicious programs and reveal confidential data, such as bank credentials, one-time passwords, and user login credentials. The sole intention is to collect personal information through malicious program-based attempts embedded in URLs, emails, and website-based attempts. Notably, this proposed technique detects URL, email, and website-based phishing attacks, which will be beneficial and secure us from scam attempts. Subsequently, the data are pre-processed to identify phishing attacks using data cleaning, attribute selection, and attacks detected using machine learning techniques. Furthermore, the proposed techniques use heuristic-based machine learning to identify phishing attacks. Admittedly, 56 features are used to analyze URL phishing findings, and experimental results show that the proposed technique has a better accuracy of 97.2%. Above all, the proposed techniques for email phishing detection obtain a higher accuracy of 97.4%. In addition, the proposed technique for website phishing detection has a better accuracy of 98.1%, and 48 features are used for analysis.

## 1 Introduction

Nowadays, phishing is a widespread tactic among cybercriminals targeted at renowned organizations and government agencies. In particular, phishing attacks are used to obtain user credentials, leading to financial and personal data loss to the victim. Subsequently, the APWG recorded approximately 4 million phishing assaults in 2022, an all-time high. The annual growth rate of phishing attacks has been over 150% since the start of 2019. The Anti-Phishing Working Group (APWG) recorded 1,350,037 unique phishing attempts in Q4 of 2022. In addition, the statistics show a modest increase compared to the previous quarter, while APWG documented a record-breaking 1,270,883 phishing attacks, marking the highest quarter for phishing ever recorded by APWG. In addition, during the fourth quarter of 2022, the APWG reported increasing scam email reports from its participants and the general public. It should be noted that the APWG experienced the highest monthly total of 101,104 distinct mail issues obtained in October 2022 (APWG, 2022), which is indeed a huge threatening factor.

**Figure 1:**
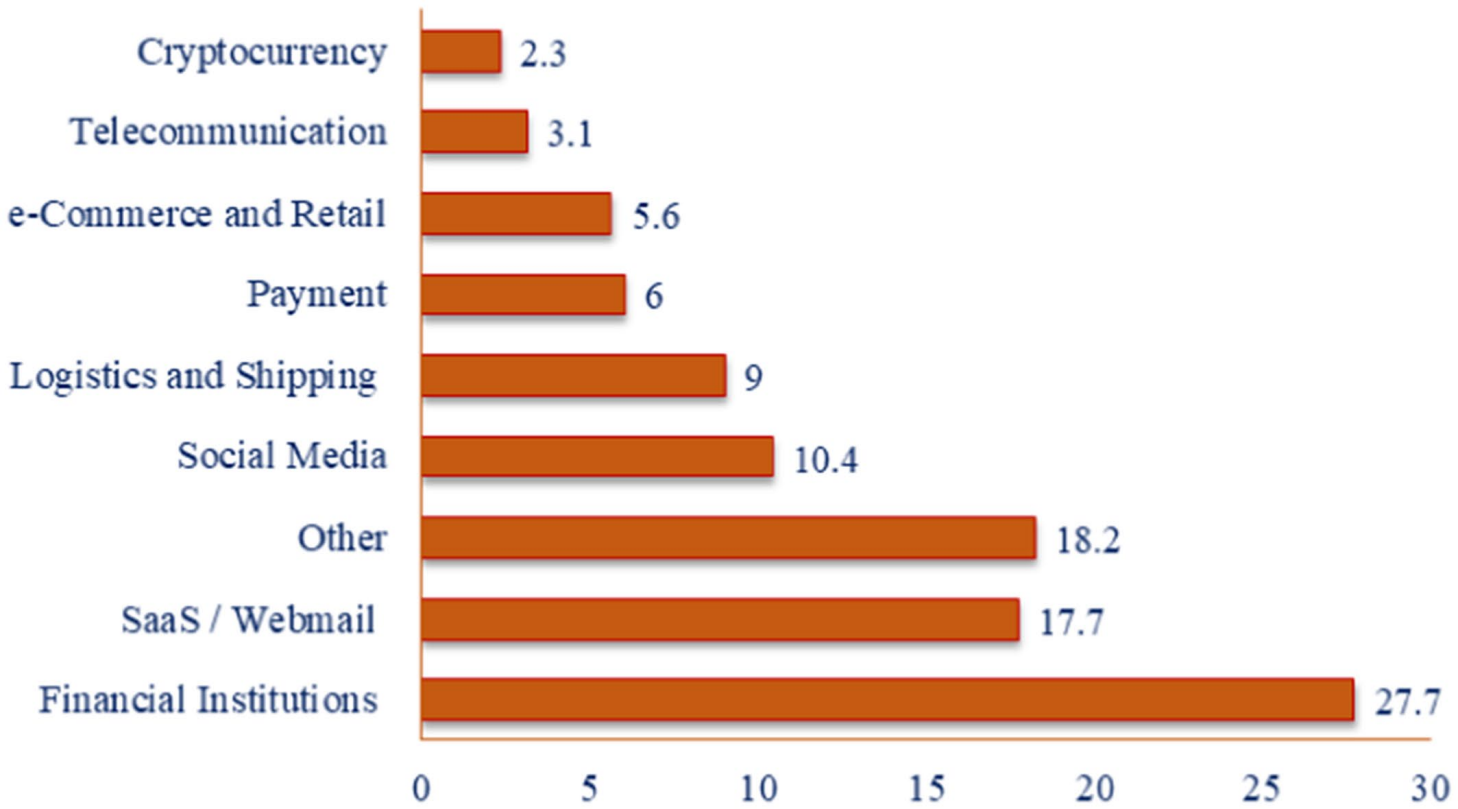
Percentage of phishing attacks on industries.

The major concern was raised when the Phishing Operation Statistics for Quarter 4 of 2022 produced by the APWG were revealed. APWG member OpSec Security discovered that phishing scams targeting the banking industry maintained the highest category of assaults in the 4th quarter of 2022. As a warning note, these attacks accounted for 27.7% of all phishing, indicating an increase over 23.2% that they accounted for in the third quarter of 2022. In the fourth quarter of 2022, assaults directed at web and mail through software as a service accounted for 17.7% of all incidents, a slight decrease compared to the previous quarter. An additional 6% comprised cyberattacks directed at transaction systems, including PayPal, Venmo, and VISA. Despite ranging from 8.5% of total assaults in the 4th quarter of 2021 to 15.5% in the 2nd quarter of 2022, phishing targeting social networking firms continued to decline. First, the plunging prices affected the cryptocurrency sector; the percentage of phishing attempts directed against cryptocurrency-related destinations, such as cryptocurrency interactions and wallet suppliers, reduced from 4.5% in the 2nd quarter to 2.0% in the 3rd quarter and 2.3% in the 4th quarter (Kheddar and Himeur, 2023). Phishing attacks on various industries are projected in Figure 1. Financial institutions faced the highest phishing attacks, 27.7%, among other industries.

The typical Business Email Compromise (BEC) assault made an effort to extract $132,559 worth of goods. In addition, the 4th Quarter report on BEC case out methods projects 39% of attacks based on phishing mail, 31% of attacks based on gift cards, 12% of attacks based on payment, 11% of attacks based on other attacks, 6% of attacks based on extortion, and 1% of attacks based on wire transfer (Demmese et al., 2024). For instance, 60% of the con artists surveyed said that Amazon gift cards were their preferred settlement method, making Amazon the clear front-runner in this category. The gift card options made available by Apple came in at a close second, with 9% of respondents wanting iTunes vouchers and 9% seeking Apple store vouchers (APWG, 2022).

**Figure 2:**
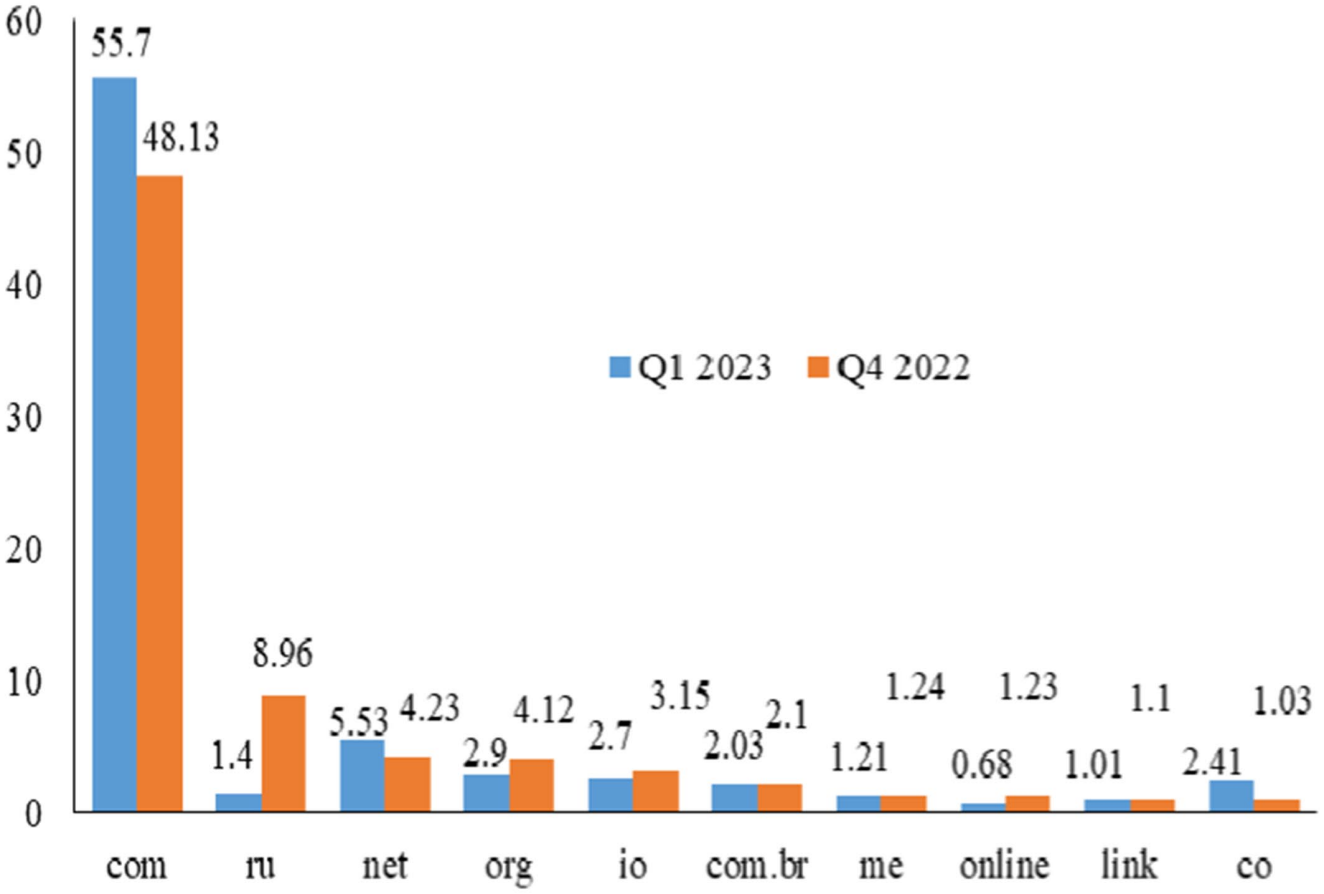
Percentage of phishing attacks during Q1 2023 and Q4 2022.

Third, according to Cofense’s Q1 2023 Intelligence Trends Review findings, expose loaders are still the majority of often used equipment for phishing attacks. It is noteworthy that key loggers and data stealers earned 2nd and 3rd place, given that roughly 74% of phishing attempts in 2019 comprised identity phishing, such as user credentials (Ma et al., 2024). In the 1st quarter of 2023, the number of mysterious harmful campaigns that abused Telegram bots continued to skyrocket, exceeding the volume of the 4th quarter of 2022 by 397% and the quantity of the whole year 2022 by 310% utilization of Web3 technology for malicious purposes, namely, as a link-crafting tool for phishing operations. For this reason, the domain-based phishing attacks are illustrated in Figure 2, and the com domain faced the highest phishing attacks of 55.7 in Q1 2023 and 48.13 in Q4 2022 and then other domains (Cofense, 2023).

Fourth, as per Zscaler’s Threat Labs 2023 Phishing Statement findings, AI technologies such as ChatGPT can generate false account pages with just a small amount of programming skill. Furthermore, it shows that this service might be used in the production of multifaceted malware and various kinds of dangerous malware. In addition, humans can employ AI-driven technologies to identify fraudulent connections (Rashid et al., 2024). However, there remains an extended way to go about a secure list. It discovered that ChatGPT-3 could identify phishing URLs 87.2% of the time. However, it resulted in a false-positive rate of 23.2%, which renders it functionally worthless unless further enhanced.

**Figure 3:**
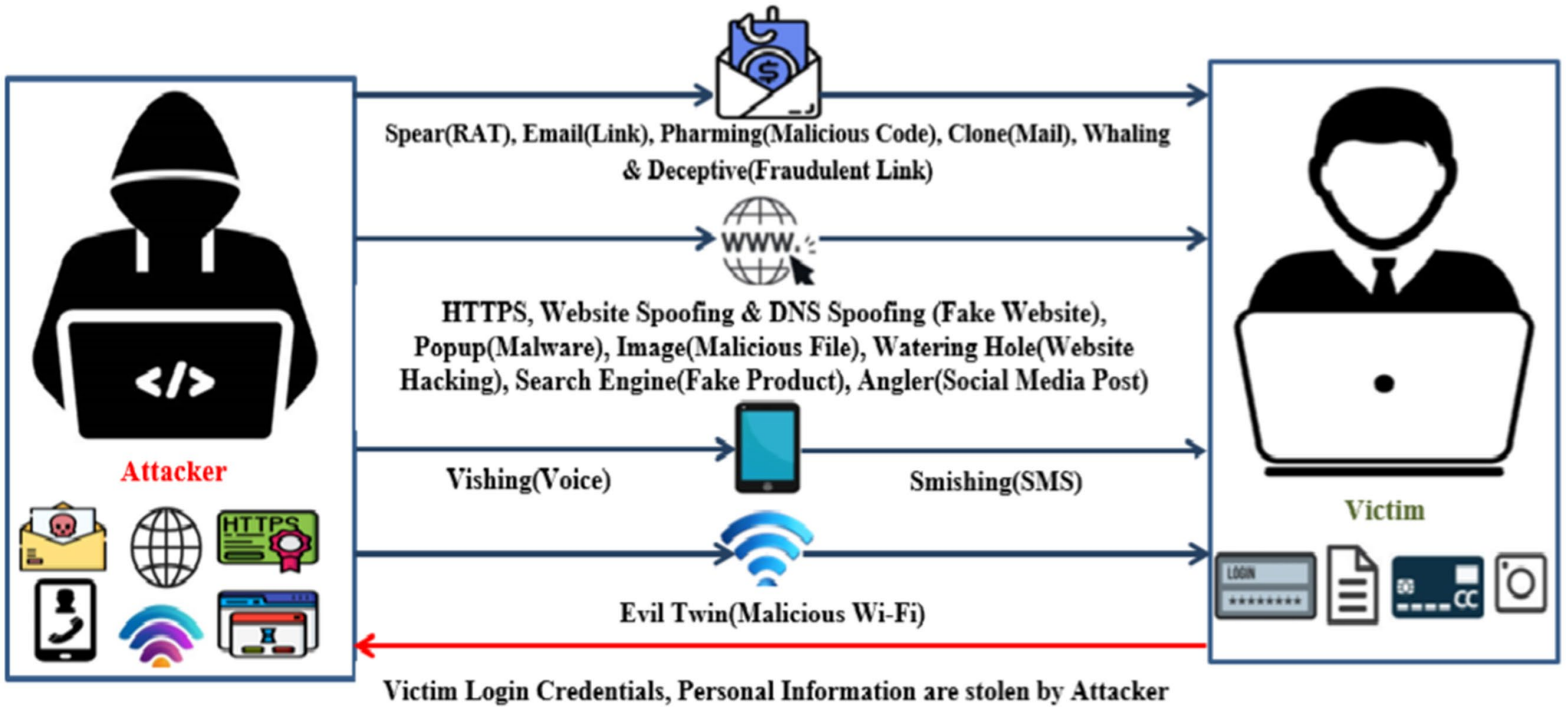
Phishing attack types.

Moreover, the phishing attacks are classified into 19 types, as shown in Figure 3. Above all, 19 types of phishing attacks are grouped into four categories: email, web, mobile, and Wi-Fi. Email-based attacks are triggered using URLs, malicious files, and RAT files (Trad and Chehab, 2024). Web-based attacks are initiated using fake websites, malicious files, and websites altered by hacking and social media attacks. The mobile-based attacks are initiated using voice calls and SMS alongside, Wi-Fi-based triggered by providing free Wi-Fi. Machine learning techniques are used to detect phishing attacks and lead to better mitigation services (ThreatLabz, 2023).

The proposed technique detects and mitigates three phishing attacks (URL, email and website). The study is structured so that Section 2 depicts a literature review on phishing attacks using email, website, URL, and Wi-Fi-based attacks. Next, Section 3 provides the methodology for URL, email, SMS, and website phishing attack detection and mitigations. Subsequently, Section 4 contains experimental results and discussion. Finally, Section 5 contains the study conclusion and future work directions.

## 2 Related works

The term Cyberspace means a variety of threats and attacks (Shaukat et al., 2020) which is an ongoing challenge encountered by the digital space. Some of the usual threats to digital space include malicious URLs, phishing attack URLs, email phishing attacks, website phishing attacks, and disabling firewalls and antivirus software (Shaukat et al., 2020; Shaukat et al., 2020). The URL phishing dataset (Hannousse, 2021) comprises 11,430 URLs, each with 87 selected characteristics. In addition, the email dataset (Mark et al., 1999) has 5,172 tuples, the SMS spam database (Tiago and Jos, 2012) has 5574 tuples, and the website phishing dataset (Tan, 2018) includes 48 characteristics that were selected from a total of 5,000 websites and are used as inputs for proposed systems.

There are few solutions or preventive measures available to address this issue. For example, understanding contemporary email phishing tactics and patterns might help create preventative policies. To further emphasize, this study (Hoheisel et al., 2023) examines how the COVID-19 epidemic affected phishing emails that did not address websites and emails generated before the COVID-19 pandemic. Furthermore, the email information is examined to see how the global epidemic affects phishing email subjects, if messages correspond with COVID-19 outbreak occurrences and patterns, and if any concealed material is disclosed. During the epidemic, 500,000 phishing emails to Dutch-authorized domains were analyzed. The research found that many COVID-19-related scam emails follow recognized trends, suggesting offenders adapt rather than innovate (Hoheisel et al., 2023). In addition, phishing attacks on transport layer security and encrypted traffic without decryption are detected using ML techniques (Kumar et al., 2023), and the phishing websites hosted detection using TWSVM techniques (Rao et al., 2021). Therefore, using a heuristic-based machine learning technique, the proposed technique detects and mitigates three types of phishing attacks (URL, email, and website).

On a special note, phishing emails have dominated cybercrime for years. Over many years, various detectors have concentrated on messages or languages. The present study offers HELPED, a phishing email identification system that employs ensemble training and hybrid characteristics. Furthermore, hybrid features accurately portray emails by combining text and words. HELPED uses stacking ensemble training and soft voting ensemble training. The hybrid attributes using ensemble training outperform content or text feature-based detection testing. As a result, soft voting ensemble training surpasses other popular machine learning methods on a rich, unbalanced dataset that tracks phishing email development (Bountakas and Xenakis, 2023), and domain-based attack detection needs to be addressed.

Depending on the input, anomaly identification methods are usually supervised, unsupervised, or semi-supervised (Shaukat et al., 2020). Thus, supervised anomaly identification is a classification issue that distinguishes abnormality from routine (Shaukat et al., 2021). Unsupervised anomaly identification needs dataset labels. It finds anomalies by understanding data instance trends and outliers (Khan and Rehman, 2023). In addition, semi-supervised anomaly identification establishes normalcy or variation for anomaly identification using normal or abnormal samples (Shaukat et al., 2021). Watermarking-based image data security is addressed (Sahu et al., 2023; Sahu et al., 2023; Sahu, 2022).

**Figure 4:**
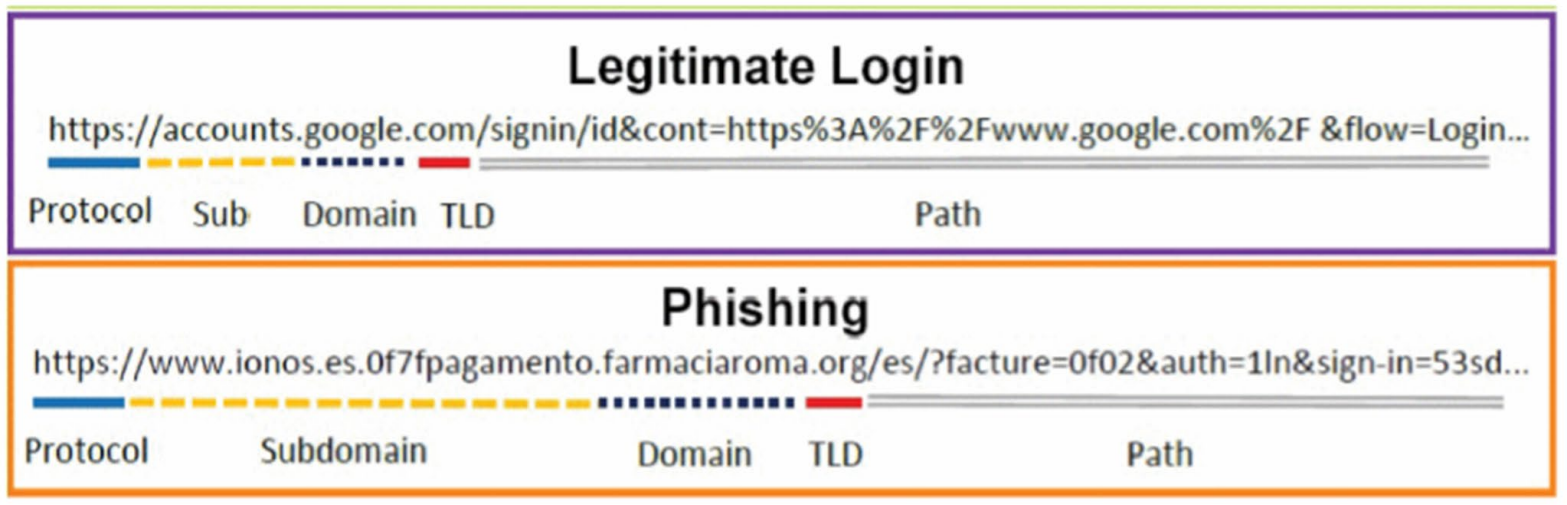
Legitimate and phishing URLs.

One of the most popular forms of phishing is URL phishing, which steals data from users who visit rogue websites. Harmful URL detection is difficult (Tariq et al., 2023). This study uses machine learning techniques to recognize websites based on the recommended URL’s behaviors and features. Online security experts block malicious websites. Denylists are built using manual reporting and site assessment criteria (Hasane Ahammad et al., 2022), and email-based attack detection is not considered. Machine learning models are employed to identify dangerous URLs. A legitimate URL contains the correct domain, subdomain, TDL, and path, and a phishing URL contains a modified URL (Sánchez-Paniagua et al., 2022), as shown in Figure 4.

This research uses convolutional neural network (CNN) fusion, a phishing URL detection approach that is both efficient and not resource intensive. CNN fusion is created on a character-level CNN. Thus, observing discrepancies between phishing and safe URLs could display a substantial geographical connection. They implement a max-over-time grouping strategy on the attribute group to choose the most significant characteristics to censor downcasts on the possibility of mistakes that irrelevant or noisy characteristics trigger force. As a solution, the proposed approach helps us to limit the likelihood of errors occurring. The last step of the evaluation of the model uses 5 datasets that are accessible to the public and together comprise 1.85 million phishing and safe URLs (Hussain et al., 2023). Any machine learning models for phishing detection must be accompanied by rigorous ethical considerations around privacy, transparency, and consent, as well as adherence to legal frameworks of General Data Protection Regulation (GDPR). A summary of phishing attack detection and its techniques is given in Table 1.

**Table 1:** Summary of phishing attack detection.

Paper	Year of published	Attacks types	Details	Algorithms and techniques used
3	2023	Email	Business email compromise with phishing attacks is detected using machine learning algorithms.	Naive Bayes, support vector machines, and logistic regression
8	2023	Email	Identification and mitigation of phishing emails using classification using deep learning.	Recurrent neural network
10	2022	URL	It uses a machine learning algorithm to detect URL phishing attacks in the Phishing Index Login URL (PILU-90 K) dataset.	Logistic regression
11	2022	Website	Website-based phishing datasets are detected using a classification technique.	LightGBM classifier
12	2023	URL	URL-based phishing attacks are detected using a one-layer convolutional neural network.	Convolutional neural network
14	2023	Email	Detection of phishing content and textual information in emails using machine learning algorithms	Ensemble learning
17	2023	Email	Email phishing detection using content and top-level domain analysis using unsupervised learning.	Clustering
19	2022	URL	URL classification into malicious or not using machine learning techniques.	Random forests, decision trees, and support vector machine
23	2020	Email, website, and URL	Email, website, and URL phishing attacks are detected using the PhishBench tool.	Classification techniques
24	2023	URL	URL phishing detection system through hybrid machine learning.	Logistic regression, support vector machine, and decision tree

### 2.1 Phishing through social engineering attacks

**Figure 5:**
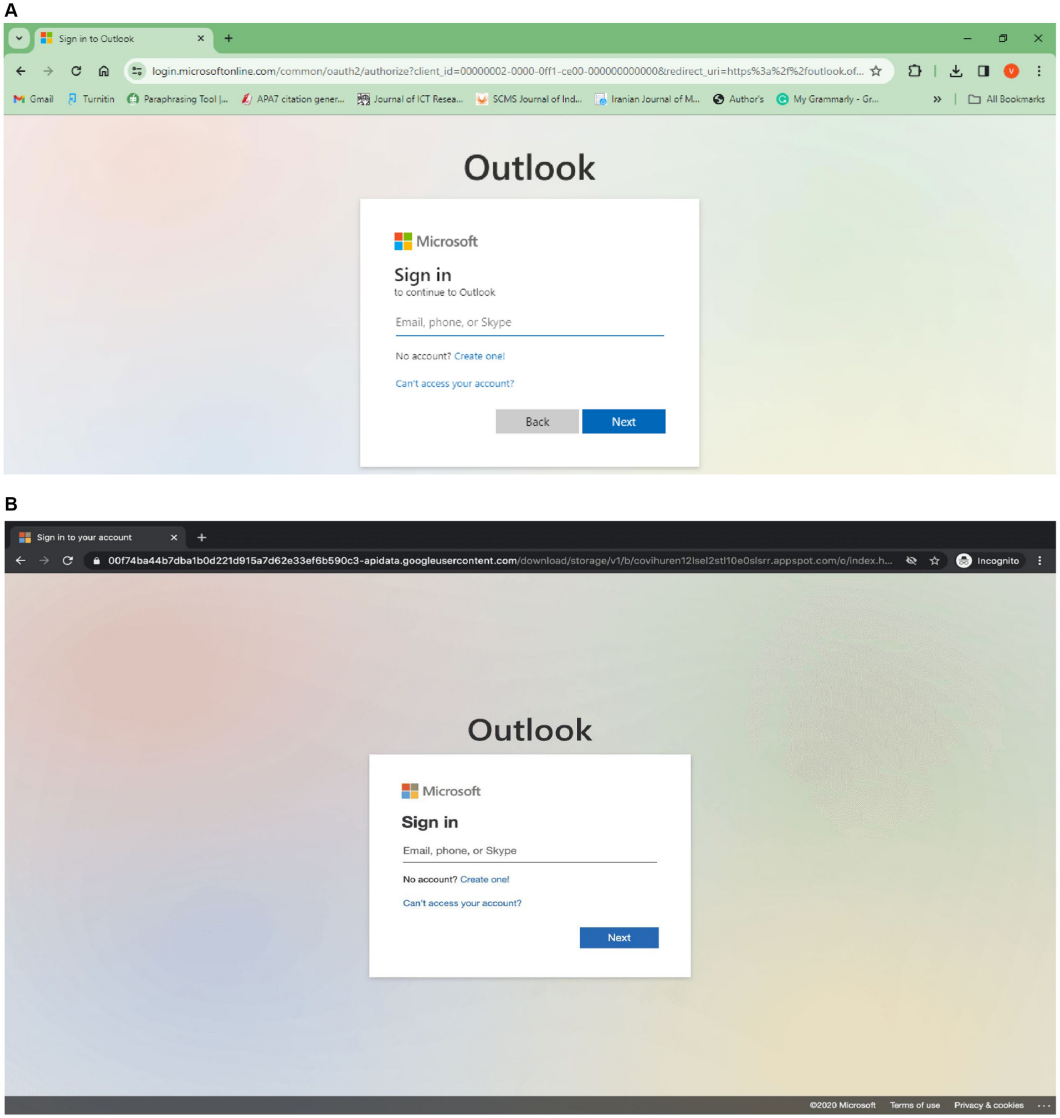
(A) Legitimate webpage. (B) Phishing webpage.

An array of cyberattacks sending a fraudulent email to an unknowing victim stands first of several cyberattacks, referred to as spear phishing. To create these emails (Atlam and Oluwatimilehin, 2023), hackers need to thoroughly study the people they target and acquire sensitive data concerning the targets and data concerning their targets’ friends and colleagues. Even though spear-phishing attempts are quite common, very little is known about the human components leading to attacks. Thus, gaining exposure to more sensitive data on victims might lead to assaults that include reports and impersonation that are socially significant. Hence, the findings of this study have important repercussions for the development of anti-phishing systems that make use of machine learning methods. The legitimate webpage is shown in Figure 5A, and a phishing webpage (Sánchez-Paniagua et al., 2022) containing a modified URL is shown in Figure 5B, in this study, favicon and SSL were not considered during attack detection.

The suggested approach eliminates the need for complex character development activities and domain expertise by fusing machine learning with deep learning. The suggested technique and its identification efficacy are verified using 15 deep learning techniques and 12 machine learning techniques. The methodology may benefit the military sector, which will aid in developing better effective malware identification programs (Shaukat et al., 2023). The deep learning models used for phishing attack detection are CNN and RNN, respectively. CNN is useful for phishing identification depending on images. RNN is good for linear data such as URL patterns and email messages.

Moreover, machine learning uses techniques such as random forests, gradient boosting machines, and support vector machines to process individual characteristics. The feature fusion integrates characteristics from machine learning and deep learning techniques. They combine the characteristic vectors along the two streams (Tianhao et al., 2023). The ensemble methods combine deep learning and machine learning projections into an ensemble model. Initially, you should use deep learning technology to exclude apparent phishing emails. After that, you should use machine learning technology to complete the classification process.

**Figure 6:**
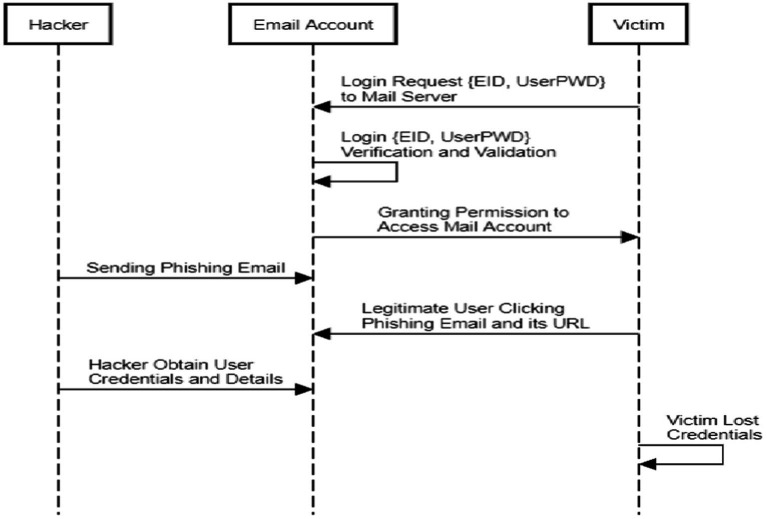
Summary of email phishing.

Foremost, a unique technique is established to construct a malicious software detector by learning a neural network using a combination of multiple adversarial attempts. This approach considers the features of multiple adversarial attacks and makes utilization of the effectiveness of the 10 reporters on various methods of evasion while designing the malware scanner (Shaukat et al., 2022). Out of the massive number of businesses engaged in trades via the Internet and offerings, phishing assaults are the most challenging social engineering interruptions. Criminals employ a login form that copycats the website to steal usernames and passwords and send them to a malicious server. Furthermore, this study detects phishing using URL, HTML, and web technologies. The Phishing Index Login Websites Dataset (PILWD) provides investigators with 134,000 validated offline phishing instances to investigate and evaluate various methods. The email-based credential theft is projected in Figure 6. On PILWD’s database, a LightGBM model with all 54 characteristics can identify fake websites with 97.95% accuracy (Sánchez-Paniagua et al., 2022).

The Domain Name System (DNS) maps IP addresses to unique domain names and vice versa, making it essential to the Internet infrastructure (Karim et al., 2023). The malevolent user exploits DNS faults. Furthermore, the DNS escalation and tunneling attacks are the most complicated DNS assaults. The intrusion detection systems (IDS) analyze network traffic for intrusions, including DNS incursions. This study introduces DNS Intrusion Detection (DID), a method incorporated into SNORT, a popular open-source IDS, to identify significant DNS-related assaults. By and by, new IDS signatures were added for tunneling, amplification, and DoS tools (Ramprasath et al., 2022; Ramprasath et al., 2023a,b and Ramprasath et al., 2021) to the IDS ruleset file to identify DNS-based intrusions. This method effectively detects empirical DNS assaults by different Internet tools. Thus, the DID has high discovery and minimal false-positive rates (Adiwal et al., 2023), and email-based phishing detection is not considered.

The organization loses a lot since BYOD allows advanced persistent threat (APT) attacks, notably watering hole attacks (Himeur et al., 2022). The BYOD in higher education posed the same concern. This study presents a watering hole occurrence and spear-phishing model, an assessment of these two APT variations, and a Protection Motivation Theory (PMT)-based survey design. The PLS-SEM analyzes survey results. The severity and vulnerability factors substantially explain the protection behavior component (Ismail et al., 2017).

SMS is one of the most popular cell phone functions. Despite technical advances, other messaging apps have not replaced SMS, so hackers use this SMS function to smash. Research on spam SMS detection and separation has not prevented smishing. Thus, this study used a rule-based SMS service to develop a smartphone app to identify and mitigate smishing attacks. The SMS service intercepts smartphone SMS. The rule-based machine learning model receives intercepted communications through API. The model determines whether the message is spam or ham using well-established criteria.

Moreover, the APIs provide analytical results to mobile apps. After a notice, the user chooses whether to keep the spam or ham (Akande et al., 2023). In wireless connections, unauthorized entry is a significant security risk. However, an individual tricked into linking to an unauthorized access point (AP) also encounters an unsafe situation. Evil twin (ET) is the name of the rogue AP, which is easy to set up despite people noticing. As a hijacking or scam, a hacker could listen in on the information or reroute it. In this study, a front-end evil twin identification method is suggested. The simulation outcomes demonstrated that the system could accurately determine whether ET was present by looking at the context’s RTT and matching MCS. Furthermore, the application is also straightforward because the individual using it has to get approximately the region to acquire data.

## 3 Methodology for phishing detection and mitigations

In recent years, phishing attacks have led to financial loss and identity theft (secret password credentials and personal information) for ordinary people and organizations. Subsequently, phishing attacks are triggered against individuals or organizations through malicious emails, websites, and URLs. A proposed technique is used to detect the three categories of phishing attacks mentioned above. The fundamental techniques to identify phishing attacks are data pre-processing for cleaning attribute selection and attack detection using a heuristic-based machine learning technique.

### 3.1 URL phishing dataset

URL phishing, or link manipulation, is a fraudulent activity where cybercriminals create fake websites or use misleading links to deceive individuals into providing sensitive information such as passwords, credit card details, or personal information. Moreover, the attackers often disguise these links as legitimate, making it challenging for users to recognize the deception. The dataset (Hannousse, 2021) that was supplied has 11,430 URLs, each with 87 selected characteristics. The dataset was developed to serve as a standard for phishing identification techniques dependent on ML. There are three distinct groups of characteristics: 56 are resultant from the format and syntax of URLs, 24 are resultant from the gratified of the sites that relate to them, and 7 are resultant through querying additional services. The dataset is well-balanced; it includes an equal number of phishing and genuine URLs, precisely 50% of each. Python scripts were employed for the mining of characteristics for prospective duplication or expansion, and these scripts have a connection with the dataset.

#### 3.1.1 URL pre-processing

**Figure 7:**
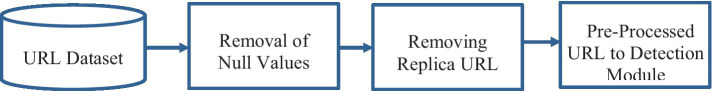
URL data pre-processing flow.

A link to an Internet resource that defines its position on an IT network and the process for getting it is referred to as a website’s address, which is short for a uniform resource locator (URL). In common parlance, a website’s address is also known as a web address. The form might be included in a standard URL http://www.sample.com/index.html, where HTTP is a protocol, www.sample.com is a hostname, and index.html is a file name. URL dataset processing is projected in Figure 7.

#### 3.1.2 Heuristic-based methods for URL phishing detection

Creating a heuristic mathematics equation for phishing URL detection involves various factors and characteristics common to phishing URLs.


(1) $$\begin{array}{l} \mathrm{Phishing}\;\mathrm{Score}=w1.f1\left(\mathrm{Length}\right)\\ +w2.f2\left(\mathrm{HTTPS}\right)+w3.f3\left(\mathrm{Domain}\;\mathrm{Age}\right)\\ +\ldots +wn.fn\left(\mathrm{other}\;\mathrm{factors}\right) \end{array}$$

URL attacks are computed using the length of the URL, HTTPS protocol, and domain age: the presence of @ symbol, subdomain depth, similarity to known brands, use of IP address, URL’s Alexa rank, and presence of suspicious words. F_i_ represents a function evaluating the ith factor, and w represents its weight, indicating its importance in Equation 1. Each factor $f_{i}$ is a function that evaluates the specific characteristic of the URL and returns a score end base. Subs are determined based on the importance and impact of each factor, often derived from statistical analysis and machine learning models trained on known phishing and benign URLs. The higher the score, the higher the probability that the URL is malicious.

### 3.2 Email and SMS phishing dataset

Email and SMS phishing are fraudulent activities that target individuals to steal information such as passwords, personal data, and bank details. The phishing links often come through unsolicited emails or messages, and unexpected communications asking the user to click on a link or provide sensitive information.

The email dataset (Mark et al., 1999) has 5,172 tuples, with one tuple representing every message with the dataset. The entire quantity of attributes is 3,002. The primary attribute encompasses the correspondent’s email ID. The name has been conventional to protect the target’s confidentiality, with more exhausting digits than the targets’ names. The last characteristic contains the estimate labels: “0” for non-spam and “1” for spam. Following the removal of non-alphabetic letters and words, the following 3,000 columns include the top 3,000 phrases that appear most often throughout all emails. The columns corresponding to the tuple that includes a specific email provide the count of each word.

Consequently, instead of being preserved in a separate file, information related to all 5,172 emails is maintained in a simplified data frame. The SMS spam database (Tiago and Jos, 2012) is an association of SMS communications which were specially labeled and gathered to research SMS spam. It includes one collection of SMS communications in English with 5,574 labeled according to whether or not they are spam or ham (legal). The documents include a single message per row. Every row comprises two sections: v1 has the label (either ham or spam), and v2 contains the actual content.

#### 3.2.1 Email and SMS pre-processing

**Figure 8:**
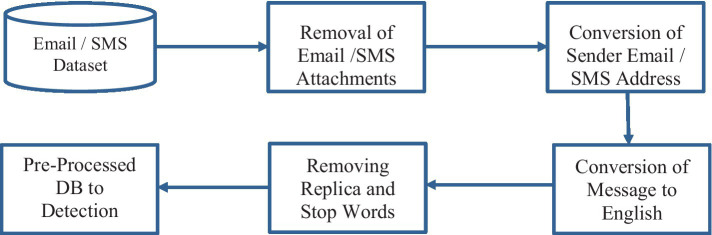
Email/SMS data pre-processing flow.

Attachments associated with emails and SMS were removed, and more focus was given on content belonging to email and SMS in this research. In future research, we will consider analyzing the email and SMS attachments. Convert the sender’s email, SMS address to a number format to maintain the sender’s privacy and convert non-English messages to English. Removing replica communication, stop words and common words from the dataset. The pre-processing module will forward the database to the phishing attack detection module, projected in Figure 8.

#### 3.2.2 Email phishing detection using sender policy framework and heuristic method

##### Algorithm 1 Email sender policy verification

**Table:** 

1	function isSPFValid(SrcIP, SrcDomain, TargetMailServerIP):
2	SPF record = queryDNSForSPF(SrcDomain)
3	If spfRecord is empty:
4	return “Neutral”
5	if sender IP is authorizedBySPF(SrcIP, SPF record, TargetMailServerIP):
6	return “Pass”
7	Else:
8	return “Fail”
9	function queryDNSForSPF(domain):
10	SPF record = query DNS(domain, “TXT”)
11	return spfRecord
12	function authorizedBySPF(SrcIP, SPF record, TargetMailServerIP):
13	if “ip4:” + senderIP in spfRecord:
14	return true
15	Else if “include:” in SPF record:
16	included domain = extractIncludedDomain(SPF record)
17	includedSPFRecord = queryDNSForSPF(included domain)
18	return authorizedBySPF(SrcIP, includedSPFRecord, TargetMailServerIP)
19	return false
20	spfResult = isSPFValid(SrcIP, senderDomain, TargetMailServerIP)
21	if spfResult == “Pass”:
22	print(“SPF validation passed”)
23	Else:
24	print(“SPF validation failed”)

Algorithm 1 represents a technique for email authentication called Sender Policy Framework (SPF) is intended to identify instances of email delivery when the sender’s (Src) address has been forged. Furthermore, SPF allows the owner of a domain to specify which mail servers they use to send mail from their domain. When targeting the mail ID, the receiver’s (Target) mail server can verify the SPF record of the sender’s domain to check whether the email comes from an authorized mail server. SPF verification is a crucial part of modern email security and is widely used to ensure that emails are authenticated and receivers are protected from phishing attacks. Algorithm 2 represents an email phishing classifier detects a given email as a phishing or legitimate email through a heuristic method.

##### Algorithm 2 Email phishing classifier

**Table:** 

S:TotalScore , $W_{x}$ : Weight of factor x , $F_{x}$ : Score from factor x analysis $F_{\mathrm{header}}$ : Analysis of the email header, $F_{\mathrm{keyword}}$ : Detection of suspicious keywords, $F_{\mathrm{link}}$ : Analysis of links and domains, $F_{\mathrm{attachment}}$ : Analysis of attachment, $F_{\mathrm{behaviou}}$ : Behavioral patterns of the sender, $F_{\mathrm{language}}$ : Language and style analysis	
1	Set S = 0
2	For each component x , analyse the email and assign a score $F_{x}$ based on the findings.
3	Each factor x has a predefined weight $W_{x}$ associated with it, reflecting its importance in determining the legitimacy of an email.
4	$S=W_{\mathrm{header}}.F_{\mathrm{header}}+W_{\mathrm{keyword}}.F_{\mathrm{keyword}}$ *+* $W_{\mathrm{link}}.F_{\mathrm{link}}$ + $W_{\mathrm{attachment}}.F_{\mathrm{attachment}}$ + $W_{\mathrm{behavior}}F_{\mathrm{behavior}}$ + $W_{\mathrm{language}}F_{\mathrm{language}}$
5	Spam Threshold: $T_{\mathrm{spam}}$ , Phishing Threshold: $T_{\mathrm{phishing}}$ , Legitimate Threshold: $T_{\mathrm{legit}}$ $\mathrm{IF}\;S>T_{\mathrm{spam}},\mathrm{then}$ *Email as Spam* $\mathrm{IF}\;S>T_{\mathrm{phishing}}$ && $S\leq T_{\mathrm{spam}}\;\mathrm{then}$ *Email as Phishing* $\mathrm{IF}\;S\leq T_{\mathrm{legit}},\mathrm{then}$ *email as l*egitimate

### 3.3 Website phishing dataset

Phishers might create subdomains that mimic legitimate sites; one must verify the primary domain and look for unusual subdomains. Admittedly, websites are cloned to target individuals who steal passwords, personal data, bank details, etc. This dataset (Tan, 2018) includes 48 characteristics that were selected from a total of 5,000 websites, 50% of which were classified as phishing websites and 50% of which were valid websites. These websites were obtained from January 2015 to May 2015 and between May 2017 and June 2017. Relative to a parsing strategy that depends on regular expressions, the enhanced approach for characteristic obtaining implemented by a web browser robotics platform (i.e., Selenium WebDriver) is superior in precision and resiliency. A Python program and GNU Wget extract all web pages and related files. It is done with the aim of accurate rendering, while the browser is not connected to the Internet. Selenium WebDriver and Python programs were employed to guide the web browser in opening the website, rendering the website’s content, obtaining the characteristic value, and storing the resultant files to automate the procedure for obtaining features.

#### 3.3.1 Website pre processing

**Figure 9:**
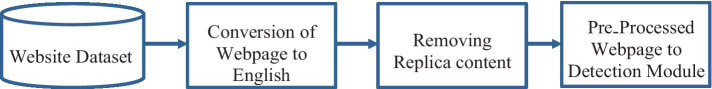
Website data pre-processing flow.

Building a website to fool users into believing another individual or company produced the web page is called website spoofing. In most cases, the fake webpage will imitate the visual appearance of the actual website it is imitating, and in some instances, the web address will be similar. Website dataset processing is illustrated in Figure 9.

#### 3.3.2 Website phishing detection using heuristic method

Algorithm 3 represents a website phishing classifier detects whether a website is a phishing or legitimate website. A website is classified as a phishing site if its phishing probability score exceeds the benchmark score.

##### Algorithm 3 Website phishing classifier

**Table:** 

P:TotalPhishingProbabilityScore , $W_{i}$ *: Weight of the i^th^ factor,* $F_{i}$ *: Score from the i^th^ factor analysis,* $F_{url}$ *: URL Analysis,* $F_{\mathrm{https}}$ *: Use of HTTPS and SSL certificate validity,* $F_{\mathrm{domain}}$ *: Domain registration length and age,* $F_{\mathrm{request}}$ *: Number of external objects requested,* $F_{\mathrm{anchor}}$ *: Anchor tags pointing to external domains,* $F_{\mathrm{forms}}$ *: Presence of forms sending data to external domains,* $F_{\mathrm{favicon}}$ *: Favicon consistency with domain,* $F_{\mathrm{content}}$ *: Content Quality and Styling,* $F_{\mathrm{popularity}}$ *: Website traffic and popularity,* $F_{TLD}:$ *: Suspicious Top-Level Domains (TLDs),* $F_{\mathrm{brand}}$ *: Brand name presence in the domain*	
1	Set P=0
2	For each component, I, analyze the website and assign a score $F_{i}$ based on the findings.
3	Each factor i has a predefined weight $W_{i}$ associated with it, reflecting its importance in determining the likelihood of the site being a phishing site.
4	$P=W_{url}.F_{url}+W_{\mathrm{https}}.F_{\mathrm{https}}$ *+* $W_{\mathrm{domain}}.F_{\mathrm{domain}}$ + ……. + $W_{\mathrm{brand}}.F_{\mathrm{brand}}$
5	Phishing Probability: $T_{\mathrm{phish}}$ $\mathrm{IF}\;P>T_{\mathrm{phish}},\mathrm{then}$ *the website is phishing*. Else *l*egitimate

## 4 Results and discussions

Experiments to detect phishing attacks are implemented on a PC with an Intel Core i7-12700 processor with 8GB DDR4 RAM and 1 TB HDD. scikit-learn and Python 3 software are used to implement the ML algorithms. It is necessary to assess the effectiveness of the machine learning (ML) algorithm by utilizing several different assessment factors. The findings generated by the ML techniques are shown in terms of projected outcomes. Regarding genuine and phishing categories, the assessment characteristics determine the number of accurate and inaccurate projections the algorithm has produced. Accuracy, precision, recall, specificity, and the F1-score were some essential characteristics employed. General Data Protection Regulation (GDPR) is used for data protection such as privacy, transparency, and consent of end user data. As demonstrated in the Equation 2, system accuracy is the percentage of correct predictions.


(2) $$\mathrm{Accuracy}=\frac{\left(TP+TN\right)}{\left(TP+TN+FP+FN\right)}$$

Primarily, this study aims to project the positive class, and precision is the assessment metric employed to study the algorithms. The precision determines the number of times the model stays right, as in Equation 3. Precision is utilized to determine the accuracy of the algorithms. Precision signifies whether the algorithm accurately predicts TP values and reflects how the model correctly categorizes phishing. Precision is measured against the optimistic accuracy of the algorithm.


(3) $$\mathrm{Precision}=\frac{TP}{\left(TP+FP\right)}$$

A measurement employed to analyze classification algorithms responds to how frequently the algorithm correctly detects possible positive classifications. Equation 4 gives this measurement to identify phishing accurately.


(4) $$\mathrm{Recall}=\frac{TP}{\left(TP+FN\right)}$$

Equation 5 shows that the F1-score is the harmonic mean of the accuracy and recall scores at the point where it achieves its highest possible value.


(5) $$F1\;\mathrm{Score}=2*\frac{\left(\mathrm{Precision}*\mathrm{Recall}\right)}{\left(\mathrm{Precision}+\mathrm{Recall}\right)}$$

### 4.1 Assessment of URL phishing finding

This experimentation comprises the assessment of 4 ML algorithms using the dataset (Hannousse, 2021). The provided dataset comprises 11,430 URLs, with 87 selected characteristics. Three distinct categories of attributes can be identified: 26 are obtained from the syntax and format of URLs, 24 are obtained from the content of the websites that are associated with them, and seven are obtained by querying additional services. The dataset is evenly distributed, including 50% phishing and 50% legal URLs. The proposed technique has a better accuracy of 97.2 compared to random forest, SVM and Naïve Bayes on URL phishing detection, which is given in Table 2.

**Table 2:** Performance analysis of URL phishing attacks.

Algorithm	Accuracy	Precision	Recall	F1-score
Proposed technique	97.2	0.98	0.97	0.97
Random forest	95.3	0.94	0.95	0.95
SVM	93.9	0.93	0.93	0.94
Naïve Bayes	89.8	0.89	0.90	0.89

### 4.2 Assessment of email phishing finding

There are 5,172 tuples in the email dataset (Mark et al., 1999), each for every message included. The total number of qualities present is 3,002. The number of times each phrase occurs in a specific email is recorded in the columns corresponding to the tuple that includes that email. This tuple contains the email in question. The sample is divided uniformly, with precisely 50% of the emails being legitimate and 50% phishing. Table 3 shows that the proposed technique has a better accuracy of 97.4 compared to random forest, SVM, and Naïve Bayes on email phishing detection.

**Table 3:** Email phishing attacks performance analysis.

Algorithm	Accuracy	Precision	Recall	F1-score
Proposed technique	97.4	0.98	0.98	0.97
Random forest	96.1	0.95	0.96	0.96
SVM	93.8	0.93	0.94	0.94
Naïve Bayes	89.6	0.89	0.89	0.90

### 4.3 Assessment of website phishing finding

The 48 features that comprise this dataset (Tan, 2018) were chosen from a total of 5,000 websites. Of these websites, 50% were identified as phishing, while the remaining 50% were considered legitimate. GNU Wget and a Python program extract all the web pages and the associated files. Table 4 shows that the proposed technique has a better accuracy of 98.1 compared to random forest, SVM, and Naïve Bayes on website phishing detection.

**Table 4:** Website phishing attack performance analysis.

Algorithm	Accuracy	Precision	Recall	F1-score
Proposed technique	98.1	0.98	0.97	0.98
Random forest	95.3	0.95	0.96	0.96
SVM	94.2	0.94	0.93	0.93
Naïve Bayes	90.1	0.89	0.90	0.91

### 4.4 Phishing accuracy models

**Figure 10:**
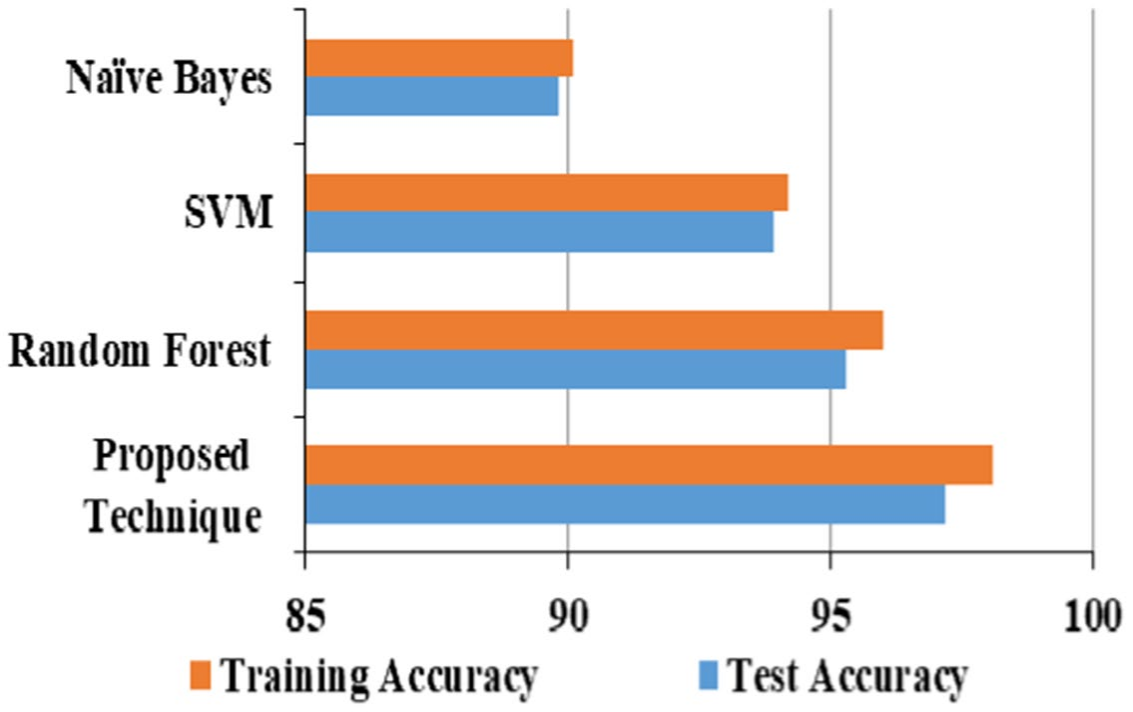
URL phishing accuracy model.

**Figure 11:**
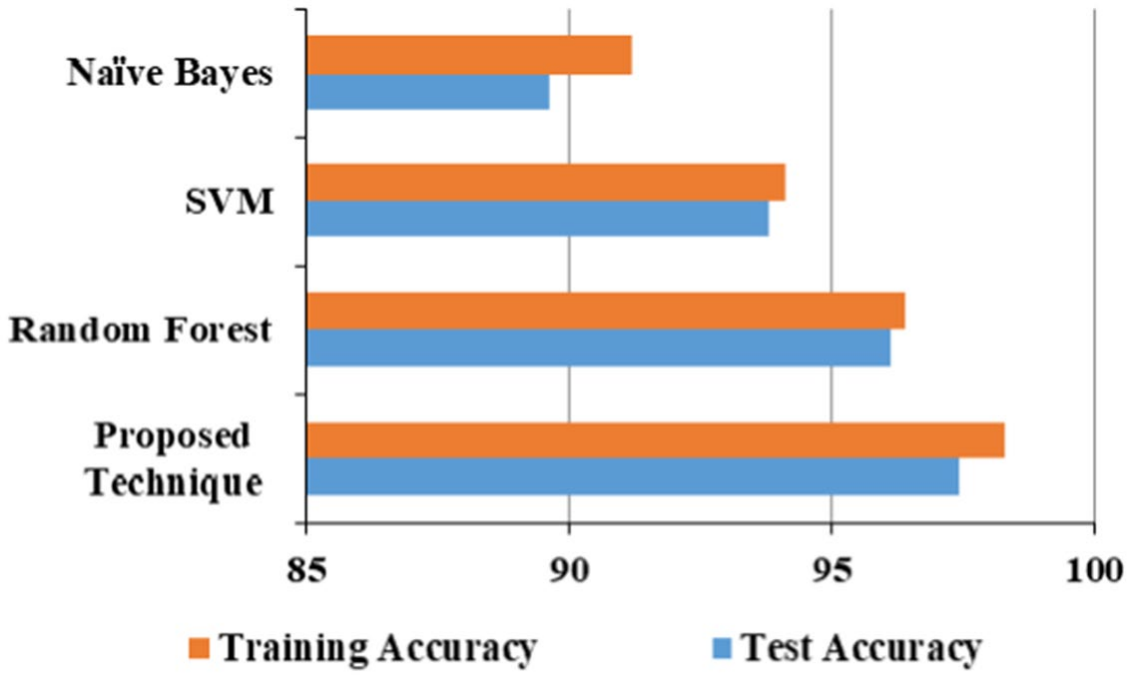
Email phishing accuracy model.

**Figure 12:**
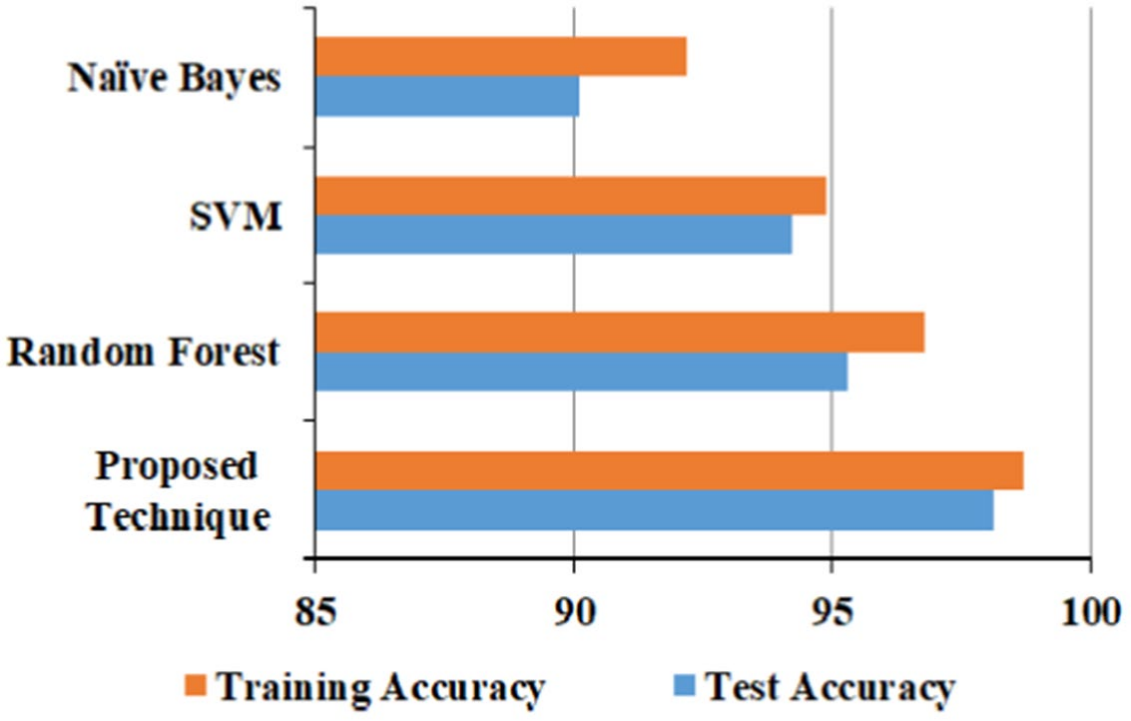
Website phishing accuracy model.

The proposed technique obtained better accuracy for the test and training model for the URL phishing dataset (Hannousse, 2021), shown in Figure 10. The email phishing accuracy model using the dataset (Mark et al., 1999) in Figure 11 projects higher accuracy on the proposed technique than other techniques. Website phishing using the dataset (Tan, 2018) has obtained better accuracy for the proposed technique training, and the test model is shown in Figure 12.

**Figure 13:**
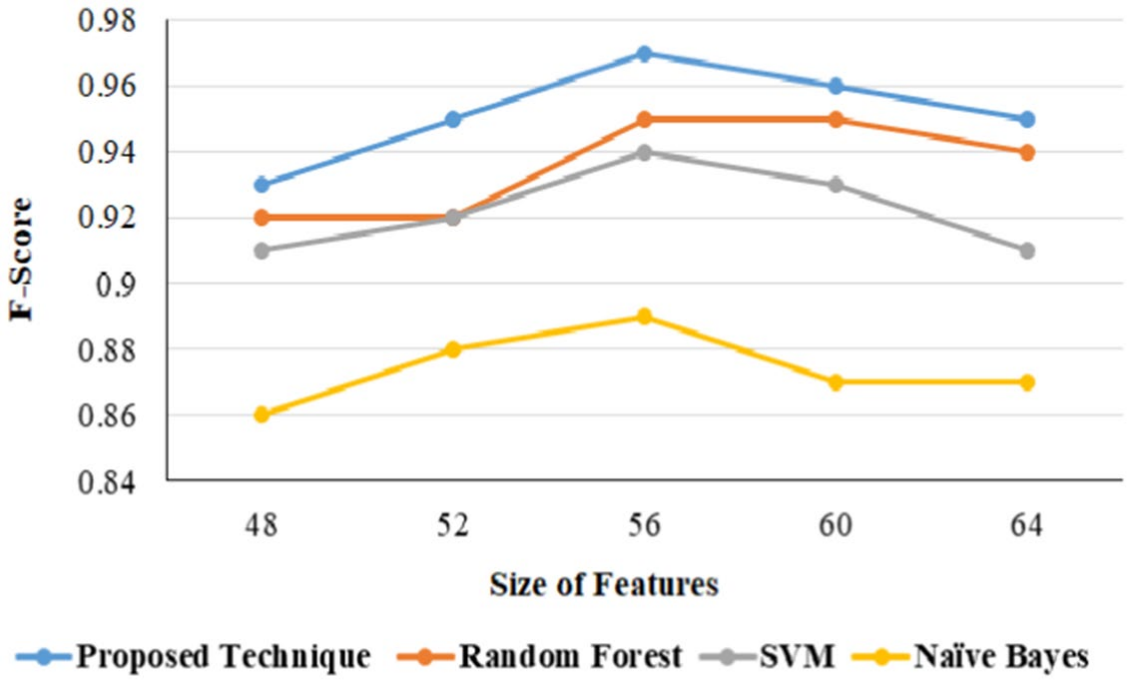
URL phishing F-score based on features.

**Figure 14:**
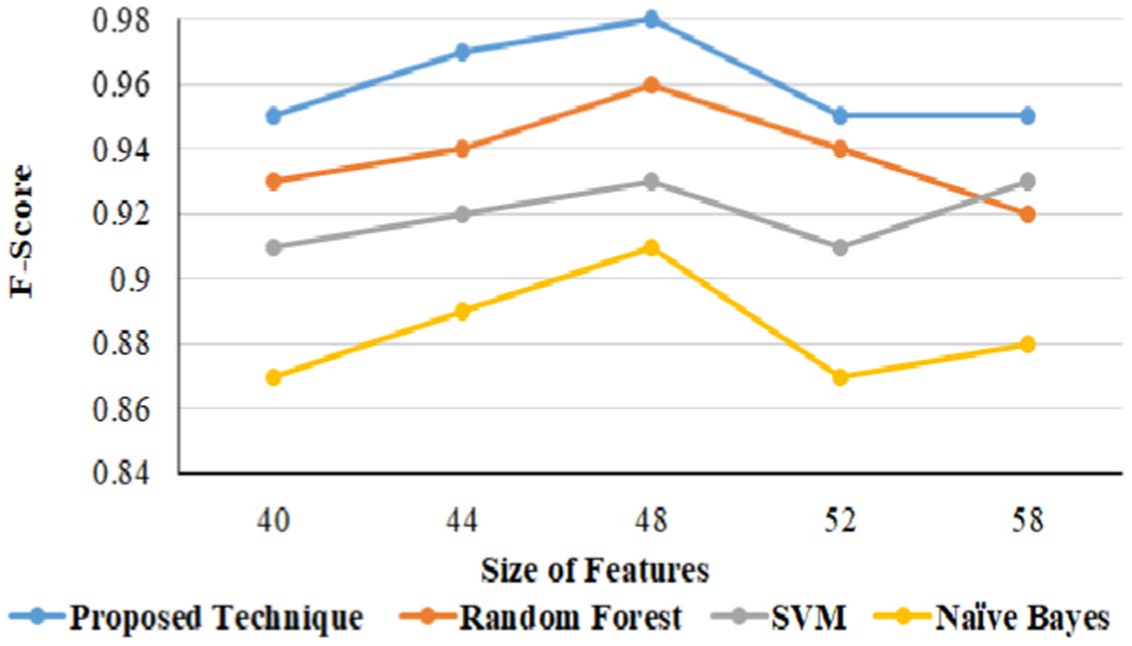
Website phishing F-score based on features.

In URL phishing detection, the proposed technique produced a better F-score of 0.97 than other techniques, and 56 features produced a higher F-score for all the models using the URL phishing dataset (Hannousse, 2021), as projected in Figure 13. The proposed technique has obtained a high F-score of 0.98, projected in Figure 14, compared to other techniques on website phishing detection using datasets (Tan, 2018). URL phishing attack detection and its performances are projected in Table 5. The proposed technique produces better accuracy on different datasets: Mendeley (Hannousse, 2021) 2021, PWD 2016, 1 M-PD 2017 (Sánchez-Paniagua et al., 2022), PIU-60 K 2020, and Ebbu 2017 (Ariyadasa et al., 2021).

**Table 5:** Proposed technique performances on URL phishing attacks.

Proposed technique performances	Mendeley (Hannousse, 2021)	PWD2016	1 M-PD	PIU-60 K	Ebbu2017
Accuracy	97.2	96.9	97.5	95.4	94.2
Precision	0.98	0.97	0.97	0.96	0.95
Recall	0.97	0.96	0.96	0.95	0.95
F1-score	0.97	0.96	0.95	0.95	0.94

Table 6 illustrates email phishing attack detection and its performance. The proposed technique produces better accuracy on different datasets: UCI (Mark et al., 1999) 2021, Nazario 2017, SpamAssassin 2017, DNC 2020, and GI Files 2017 (Aassal et al., 2020).

**Table 6:** Proposed technique performances on email phishing attacks.

Proposed technique performances	UCI ML (Mark et al., 1999)	Nazario	SpamAssassin	DNC	GI Files
Accuracy	97.4	97.5	97.2	96.9	95.9
Precision	0.98	0.97	0.97	0.96	0.96
Recall	0.98	0.95	0.96	0.95	0.95
F1-score	0.97	0.95	0.96	0.96	0.95

Table 7 illustrates website phishing attack detection and its performance. The proposed technique produces better accuracy on different datasets: Mendeley (Tan, 2018) 2021, DANS 2021 (Ariyadasa et al., 2021), Vrbancic 2020 (Vrbančič, 2020), UCI ML 2024 (Prasad and Chandra, 2024), and OpenPhishare 2018 (Chiew et al., 2018).

**Table 7:** Proposed technique performances on website phishing attacks.

Proposed technique performances	Mendeley (Tan, 2018)	DANS	Vrbancic	UCI ML	OpenPhish
Accuracy	98.1	97.5	97.3	95.8	96.2
Precision	0.98	0.97	0.97	0.95	0.95
Recall	0.97	0.96	0.96	0.94	0.94
F1-score	0.98	0.95	0.95	0.94	0.95

**Figure 15:**
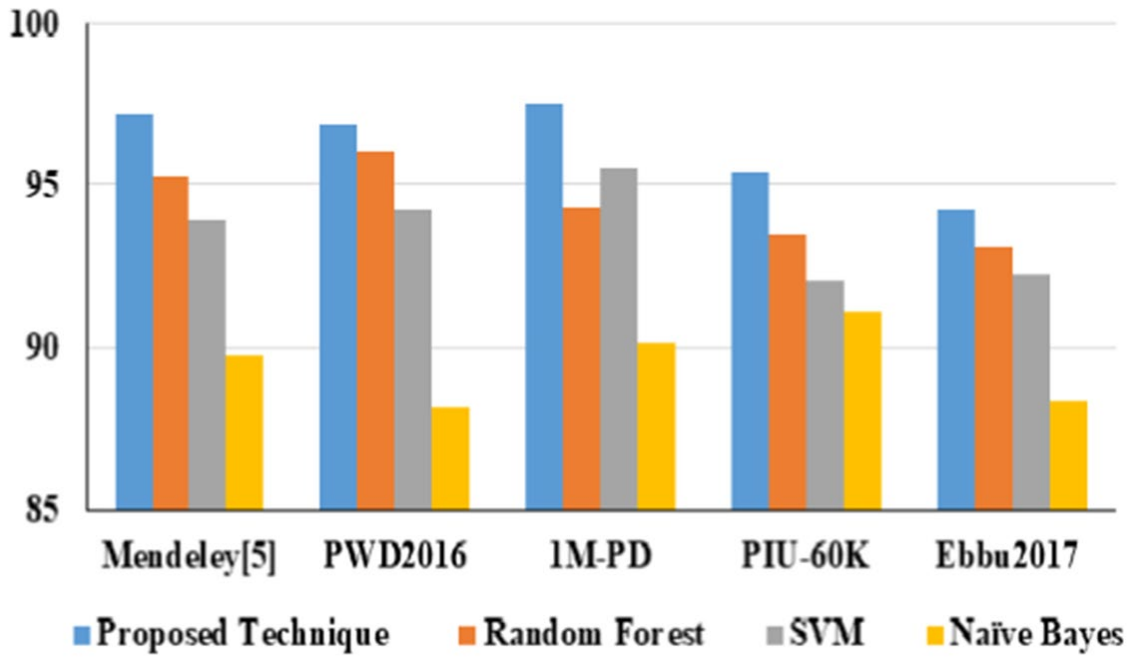
Dataset-based URL phishing accuracy.

**Figure 16:**
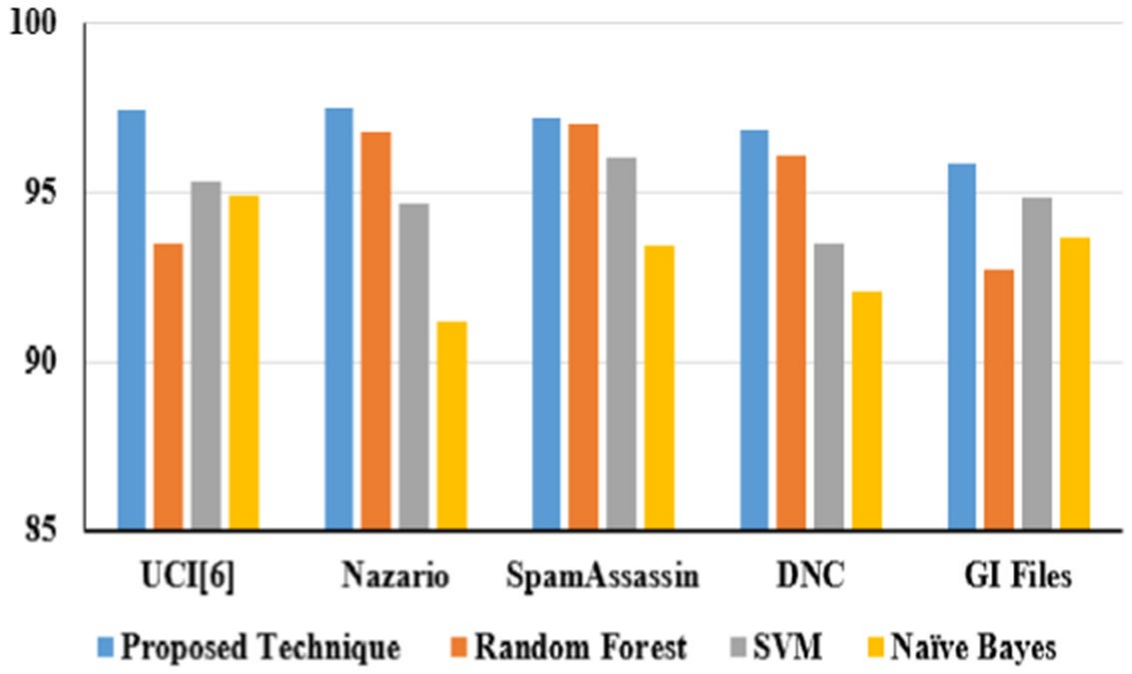
Dataset-based email phishing accuracy.

**Figure 17:**
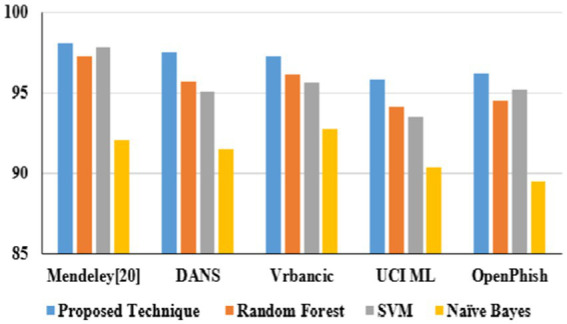
Dataset-based website phishing accuracy.

In URL phishing detection, the proposed technique obtained a higher accuracy of 97.5%, which is projected in Figure 15, and five different datasets, namely, Mendeley (Hannousse, 2021) 2021, PWD 2016, 1 M-PD 2017 (Sánchez-Paniagua et al., 2022), PIU-60 K 2020, and Ebbu 2017 (Ariyadasa et al., 2021), are used to evaluate the algorithm performances. In email phishing detection, the proposed technique obtained a higher accuracy of 97.4%, which is illustrated in Figure 16, and five different datasets, namely, UCI (Mark et al., 1999) 2021, Nazario 2017, SpamAssassin 2017, DNC 2020, and GI Files 2017 (Aassal et al., 2020), are used to evaluate the algorithm performances. In website phishing detection, the proposed technique obtained a higher accuracy of 97.5%, which is illustrated in Figure 17, and five different datasets, namely, Mendeley (Tan, 2018) 2021, DANS 2021 (Ariyadasa et al., 2021), Vrbancic 2020 (Vrbančič, 2020), UCI ML 2024 (Prasad and Chandra, 2024), and OpenPhishare 2018 (Chiew et al., 2018), are used to evaluate the algorithm performances. Mendeley, Ebbu2017, UCI ML, Vrbancic, and OpenPhish are essential for creating machine learning classifiers for phishing detection, providing an extensive database of authentic and phishing from real-world examples. A PWD (password) phishing dataset is usually made up of information that records phishing URLs intended to trick victims into stealing their login credentials. 1 M-PD contains phishing URLs, URL components, and phishing indicators that are quite beneficial for developing machine learning models that seek to identify phishing attacks instantly. Behavioral indicators, categorization, and URL features are contained in PIU-60 K dataset. A common benchmark in studies on real-time Internet user protection systems is the Nazario Dataset. The SpamAssassin datasets contains spam emails. The DNC dataset primarily involves spear-phishing attacks aimed at high-profile political figures. The GI Files Phishing Dataset refers to a collection of emails and documents associated with global intelligence company Stratfor. The DANS is a network-based phishing attacks. A one-way analysis of variance (ANOVA) was used to assess the impact of URL category (phishing vs. non-phishing) on URL characteristics. There was a statistically significant difference in the mean URL characteristics between at least two groups, according to the ANOVA in Equation 6.


(6) $$\left(F\;\left(1,N-2\right)=\left[F-\mathrm{value}\right],\;p=\left[p-\mathrm{value}\right]\right)$$

ANOVA findings show a significant difference (*p* = 0.02) in the mean domain length between phishing and non-phishing URLs. According to Tukey’s HSD test, phishing URLs often have larger domain lengths than non-phishing URLs (95% CI = [−14.48, −0.92]).

**Figure 18:**
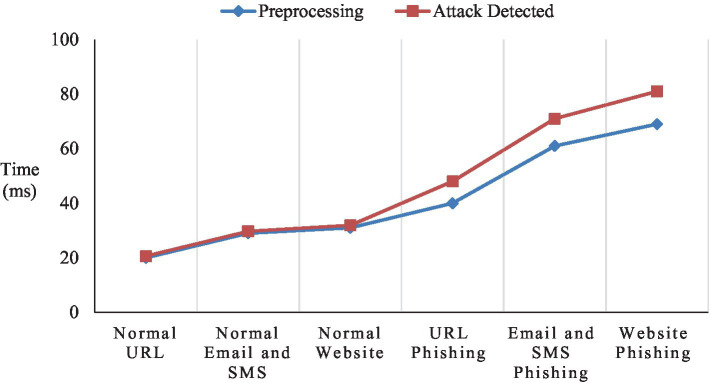
Real-time traffic types.

Quick analysis of network traffic, messages, or online traffic is necessary for real-time detection. It is difficult to guarantee identification accuracy while ensuring minimal latency, particularly considering the large amount of web activity. Real-time traffic analysis on malicious phishing attack detection faces pre-processing and attack detection delay, and it is projected in Figure 18.

## 5 Conclusion

Phishing attacks are increasingly sophisticated, leading to financial losses and identity theft in this digital world. Identifying them across different channels is crucial for user protection. Hence, this proposed study presents a framework for detecting phishing attacks across three categories: URL, email, and website. The proposed heuristic-based machine learning technique has accurately identified malicious phishing attacks under different datasets. Accuracy on the proposed technique for URL phishing detection on Mendeley is 97.2%, PWD2016 is 96.9%, 1 M-PD is 97.5%, PIU-60 K is 95.4%, and Ebbu2017 is 94.2%. Accuracy on the proposed technique for email phishing detection on UCI ML is 97.4%, Nazario is 97.5%, SpamAssassin is 97.2%, DNC is 96.9%, and GI Files is 95.9%. Accuracy on the proposed technique for website phishing attack detection on Mendeley is 98.1%, DANS is 97.5%, Vrbancic is 97.3%, UCI ML is 95.8%, and OpenPhish is 96.2%. The proposed study performs better than random forest, SVM, and Naive Bayes algorithms in all categories. Hence, this research demonstrates the effectiveness of heuristic-based machine learning in detecting phishing attacks with high accuracy across different channels. Future study could involve adapting the model to handle multimodal phishing attack detection such as image, videos, or social engineering-based phishing techniques and analyzing attachments in email and other message services.

## Data Availability

The original contributions presented in the study are included in the article/supplementary material, further inquiries can be directed to the corresponding author/s.
